# 

**Published:** 1881-05

**Authors:** 


### CONTENTS.

### COMMUNICATIONS.

Development of the Teeth, with their Arterial and Nervous Supply.. 177
Response to the Toast, "Dentistry and Dental Education—Its Past, Present and Future, as Related to Medicine........................ 184
The Education of Dentists................................................ 194

### SELECTIONS.

The Pharmacology of Chloral Hydrate.................................. 200
Effects of Electric Light on the Eye...................................... 200
The Metric System.......................................................... 201
The Anæsthetic Action of Electricity.................................. 201
Local Anæsthesia of the Larynx........................................... 202
Bacteria in the Air............................................................ 203
Medical Diplomas............................................................. 203
Go Away from Home to Learn the News................................ 204
Anatomical Material......................................................... 205
The Structure of Metals.................................................... 209
The Daily Work of the Heart............................................. 210
Substitute for Tobacco..................................................... 210
"Gas"............................................................................ 211
Legal ........................................................................... 213
Correspondence—Board of Examiners................................. 212

### EDITORIAL.

Education ...................................................................... 215
Dental Almanac................................................................ 217
Association ..................................................................... 218
Bibliographical................................................................ 219
Association ..................................................................... 220

